# Supplementary material for: Mechanism of the cadherin–catenin F-actin catch bond interaction
Source: eLife. 2022 Aug 1;11:e80130. doi: 10.7554/eLife.80130 (PMC9402232; doi:10.7554/eLife.80130)
Supplement: Supplementary file 1. [file elife-80130-supp1.docx]

|  | 6DV1 | 4IGG A | 4IGG B | 6UPV |
| --- | --- | --- | --- | --- |
| Last resolved residue | 843 | 861 | 872 | 871 |
| **RMSD, minimized with and without actin (Å)** | 0.4 | 0.6 | 0.5 | 0.6 |
| **Ca RMSD to 6UPV ABD (Å)** | 4.5 | 3.3 | 4.1 | - |
| **ABD:actin hydrogen bonds** | 11 | 7 | 13 | 16 |
| Total ABD residues | 10 | 6 | 9 | 12 |
| ABD residues (1-843) | 10 | 6 | 9 | 7 |
| CTE ABD residues (843-906) | 0 | 0 | 0 | 5 |
| ABD residues in common with 6UPV | 3 | 2 | 3 | - |
| Actin residues | 9 | 6 | 11 | 14 |
| actin residues in common with 6UPV | 3 | 1 | 3 | - |
| **ABD:actin atom distances (3.6 Å cutoff)** |  |  |  |  |
| Total ABD residues | 30 | 32 | 34 | 37 |
| ABD residues (1-843) | 30 | 32 | 31 | 26 |
| CTE ABD residues (843-906) | 0 | 0 | 3 | 11 |
| ABD residues in common with 6UPV | 19 | 20 | 25 | - |
| Actin residues | 32 | 35 | 36 | 39 |
| actin residues in common with 6UPV | 21 | 21 | 23 | - |
| **ABD:actin atom distances (4 Å cutoff)** |  |  |  |  |
| ABD residues | 36 | 36 | 36 | 39 |
| ABD residues (1-843) | 36 | 36 | 33 | 28 |
| CTE ABD residues (843-906) | 0 | 0 | 3 | 11 |
| ABD residues in common with 6UPV | 24 | 25 | 25 | - |
| Actin residues | 35 | 43 | 37 | 42 |
| actin residues in common with 6UPV | 22 | 26 | 26 | - |
| **Surface contact area (Å^2^)** | 1603 | 1768 | 1629 | 2099 |
