# Supplementary material for: Mechanism of the cadherin–catenin F-actin catch bond interaction
Source: eLife. 2022 Aug 1;11:e80130. doi: 10.7554/eLife.80130 (PMC9402232; doi:10.7554/eLife.80130)
Supplement: Supplementary file 2. — The estimated parameters for the purported weak state in the two-state slip bond model, B1, predicted binding lifetimes an order of magnitude larger than that of ternary wild type. Akaike information criterion (AIC) and Bayesian information criterion (BIC indicate the slip-bond model better describes the data). [file elife-80130-supp2.docx]

| TernaryΔH1: two-state slip bond | | |
| --- | --- | --- |
|  | B_2_ → U | B_1_ → U |
| Probability | 0.56 | 0.44* |
| CI | (0.43, 0.67) | (0.33, 0.57)* |
| $\text{k}_{\text{i→j}}^{\text{0}}$ | 0.32 | 1.17 |
| CI (s^-1^) | (0.25, 0.41) | (0.85, 1.75) |
| $\text{x}_{\text{i→j}}$ | 0.46 | 0.66 |
| CI (nm) | (0.29, 0.61) | (0.43, 0.87) |
| AIC | 12.29 | |
| BIC | 36.83 | |

**Parameters subject to P_1_+P_2_=1 constraint, and were not fit via MLE*
