# Supplementary material for: Mechanism of the cadherin–catenin F-actin catch bond interaction
Source: eLife. 2022 Aug 1;11:e80130. doi: 10.7554/eLife.80130 (PMC9402232; doi:10.7554/eLife.80130)
Supplement: Supplementary file 3. — Biexponential fit R2 are higher in both the ternary wild-type and ternaryΔH1 datasets, although the single exponential model describes ternaryΔH1 better (R2 > 0.95) than it does for ternary wild type (R2 > 0.8). [file elife-80130-supp3.docx]

| Ternary wild-type | | | | | |
| --- | --- | --- | --- | --- | --- |
|  | **N** | **Single Exponential**  **k** | **Single**  **exponential**  **R^2^** | **Biexponential**  **[k_1_, k_2_, P_1_]** | **Biexponential**  **R^2^** |
| **0-2 pN** | 12 | 6.62 | 0.807 | [42.61 1.76 0.48] | 0.896 |
| **2-4 pN** | 84 | 6.45 | 0.932 | [12.88 0.71 0.69] | 0.986 |
| **4-6 pN** | 214 | 1.50 | 0.882 | [10.81 0.58 0.38] | 0.991 |
| **6-8 pN** | 239 | 1.50 | 0.929 | [6.27 0.64 0.40] | 0.996 |
| **8-10 pN** | 106 | 2.11 | 0.961 | [3.87 0.49 0.67] | 0.996 |
| **10-12 pN** | 34 | 2.55 | 0.939 | [8.55 1.12 0.43] | 0.993 |

| TernaryΔH1 | | | | | |
| --- | --- | --- | --- | --- | --- |
|  | **N** | **Single Exponential**  **k** | **Single**  **exponential**  **R^2^** | **Biexponential**  **[k_1_, k_2_, P_1_]** | **Biexponential**  **R^2^** |
| **0-2 pN** | 69 | 0.49 | 0.975 | [0.25 1.14 0.56] | 0.994 |
| **2-4 pN** | 132 | 1.13 | 0.976 | [3.54 0.69 0.33] | 0.996 |
| **4-6 pN** | 226 | 1.32 | 0.974 | [3.15 0.64 0.46] | 0.999 |
| **6-8 pN** | 249 | 1.34 | 0.958 | [3.13 0.51 0.53] | 0.997 |
| **8-10 pN** | 151 | 1.30 | 0.984 | [4.17 0.89 0.27] | 0.998 |
| **10-12 pN** | 31 | 3.33 | 0.970 | [4.51 0.22 0.84] | 0.991 |
