# Supplementary material for: Mechanism of the cadherin–catenin F-actin catch bond interaction
Source: eLife. 2022 Aug 1;11:e80130. doi: 10.7554/eLife.80130 (PMC9402232; doi:10.7554/eLife.80130)
Supplement: Supplementary file 4. [file elife-80130-supp4.docx]

|  | **D value** | **KS statistic** | **p value** |
| --- | --- | --- | --- |
| **0-2 pN** | 0.425 | 0.703 | *1.68x10^-5^ |
| **2-4 pN** | 0.190 | 0.448 | *8.19x10^-10^ |
| **4-6 pN** | 0.130 | 0.146 | 0.016 |
| **6-8 pN** | 0.123 | 0.124 | 0.042 |
| **8-10 pN** | 0.172 | 0.175 | 0.038 |
| **10-12 pN** | 0.338 | 0.186 | 0.549 |

*Significance threshold is 0.01
