# Supplementary material for: Mechanism of the cadherin–catenin F-actin catch bond interaction
Source: eLife. 2022 Aug 1;11:e80130. doi: 10.7554/eLife.80130 (PMC9402232; doi:10.7554/eLife.80130)
Supplement: Supplementary file 5. [file elife-80130-supp5.docx]

| ABD single-step: two-state catch bond, non-directional fit | | | | |
| --- | --- | --- | --- | --- |
|  | 2 → 0 | 2 → 1 | 1 → 0 | 1 → 2 |
| $\text{k}_{\text{i→j}}^{\text{0}}$ | 0.003 | 0.94 | 2.31 | 1.71 |
| CI (s^-1^) | (0.003, 0.16) | (0.40, 1000) | (1.00, 4.74) | (0.51, 4.07) |
| $\text{x}_{\text{i→j}}$ | 1.29 | 0.65 | 0.19 | 0.09 |
| CI (nm) | (0.12, 1.45) | (0.29, 10.37) | (0.002, 0.44) | (0.004, 0.42) |

| αE-catenin monomer single-step: two-state catch bond, non-directional fit | | | | |
| --- | --- | --- | --- | --- |
|  | 2 → 0 | 2 → 1 | 1 → 0 | 1 → 2 |
| $\text{k}_{\text{i→j}}^{\text{0}}$ | 0.003 | 15.36 | 18.39 | 5.00 |
| CI (s^-1^) | (0.003, 0.44) | (4.81, 1000) | (12.09, 24.22) | (0.68, 10.09) |
| $\text{x}_{\text{i→j}}$ | 2.06 | 2.13 | 0.002 | 0.25 |
| CI (nm) | (0.008, 2.67) | (1.22, 8.17) | (0.002, 0.283) | (0.004, 1.56) |
